# Recent advances on blinatumomab for acute lymphoblastic leukemia

**DOI:** 10.1186/s40164-019-0152-y

**Published:** 2019-11-06

**Authors:** Juanjuan Zhao, Yongping Song, Delong Liu

**Affiliations:** 1 Department of Hematology, The Affiliated Cancer Hospital of Zhengzhou University and Henan Cancer Hospital, Zhengzhou, China; 2 Department of Medicine, New York Medical College and Westchester Medical Center, Valhalla, NY 10595 USA

**Keywords:** Acute lymphoblastic leukemia, Blinatumomab, BiTE, Bispecific T cell engager

## Abstract

Although complete remission rate of B cell acute lymphoblastic leukemia (B-ALL) has improved significantly over the past few decades, patients with relapsed/refractory ALL still have dismal outcome. Tyrosine kinase inhibitors, antibody–drug conjugates and chimeric antigen receptor T cell therapy are changing the therapy landscape for B- ALL. Blinatumomab, a bi-specific T cell engager, has been approved for patients with relapsed/refractory and minimal residual disease positive B-ALL. This review summarized data from recent clinical trials of blinatumomab for B-ALL treatment.

## Background

**Fig. 1:**
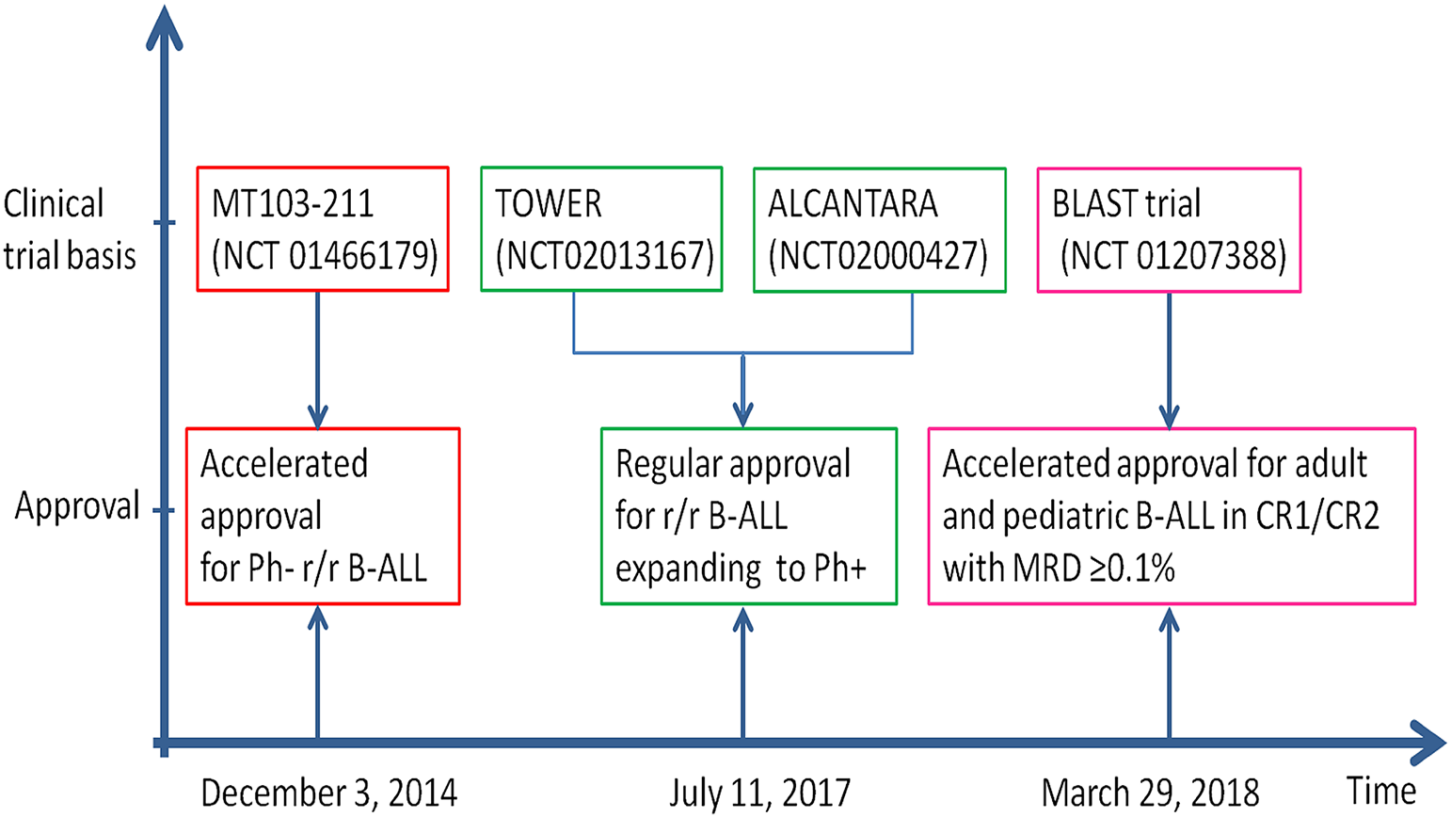
The approval timeline of blinatumomab by US FDA. *r/r* refractory/relapsed

Chemotherapy combined with targeted therapies and improved supportive care has enhanced complete remission (CR) rate of newly diagnosed B cell acute lymphoblastic leukemia (B-ALL) to 85–90%, and long-term survival rate to 40–45% [1]. However, about a third of standard-risk and two-thirds of high-risk patients experience recurrence [2, 3]. Relapsed and refractory (r/r) ALL has low rates of CR and poor long-term survival [2, 4, 5]. A retrospective analysis of 1706 r/r B-ALL patients without Philadelphia chromosome (Ph) and aged younger than 65 years showed that the CR rates were respectively 40%, 21%, 11% and 3-year survival rate were 11%, 5%, 4% for first, second, and ≥3rd salvage therapy, indicating progressively worse prognosis with each subsequent relapse [6]. Efforts are being made to improve the outcome of R/R ALL. First of all, to better define the disease status for guiding further therapy for consolidation and maintenance, minimal residual disease (MRD) is characterized more precisely with real-time quantitative PCR and multiparametric flow cytometry. MRD is defined as positive if blast cells are detected at above 0.01% level [7]. Next-generation sequencing (NGS) is increasingly used for monitoring MRD and better predicting early relapse [8, 9]. About 30–50% of adults and 10–20% of children with ALL achieved MRD- negative CR [10–14]. MRD is currently recognized as the most significant indicator for ALL relapse at all ages [15–17]. A meta-analysis of a total of 39 studies including 13,637 ALL individuals showed a significant correlation between negative MRD and 10-year EFS (HR = 0.23 for pediatric subjects and = 0.28 for adult subjects) and OS (HR = 0.28 for pediatric subjects and = 0.28 for adult subjects) [18]. Secondly, to add targeted agents such as tyrosine kinase inhibitors and CD20 antibodies to chemotherapy regimens when appropriate biomarker targets are present [19–26]. CD19 is expressed in normal and malignant B cells [27–30]. Engineered T cells with CD19- targeted chimeric antigen receptors (CAR T) are widely studied for R/R ALL [31–33]. Recently, a CD19 × CD3 bispecific T cell engager (BiTE), blinatumomab (Blincyto, Amgen), has been developed [34]. Blinatumomab contains CD3 and CD19 single-chain variable regions linked by a glycine–serine linker. It binds selectively to CD3 expressing T cells and CD19 expressing B cells, leading to the formation of immune synapses between T cells and B cells [35, 36]. This redirects unstimulated cytotoxic T cells to specifically target and lyse CD19-positive B cells, both malignant and normal B cells. The blinatumomab BiTE single-chain antibody fragment has a molecular weight of 54 kDa. Blinatumomab is administered through continuous IV infusion for 4 weeks followed by a 2-week interval [37–39]. Similar to CAR T therapy, cytokine release syndrome (CRS) and neurotoxicity are the two major adverse events associated with blinatumomab therapy [38, 40, 41]. Blinatumomab achieved accelerated US Food and Drug Administration (FDA) approval for R/R ALL (Fig. 1) [42, 43]. Blinatumomab has been approved for treatment of R/R ALL in 53 countries [44]. This review summarized recent updates on clinical trials of blinatumomab for B-ALL.

## Blinatumomab clinical trials for ALL

### Blinatumomab for R/R ALL

In a phase 2 clinical trial of blinatumomab for R/R B-ALL, a total of 36 patients were enrolled. The CR/CRh rate was 69% (25/36) after the first two cycles. Among the responders, 88% (22/25) achieved a molecular remission. The MRD-negative response rate was 69%. It was noticed that the quality of response was worse in second or greater relapse. With a median follow-up of 9.7 months (m), the median relapse-free survival (RFS) was 7.6 m [45]. Since CD19+ normal B cells were also affected, lymphopenia was the most frequent severe adverse event (SAE). After cessation of therapy, lymphopenia became reversable. After longer follow-up (median 33 months), 80% MRD-negative response rate was reported [46]. This study with long-lasting complete remission in R/R B-lineage ALL patients laid foundation for further expanded clinical investigation.

In a separate large, multicenter, phase II trial (MT103-211, NCT01466179), 189 adult patients with Ph-negative R/R B cell ALL were enrolled to further assess the clinical activity of blinatumomab [38]. Patients who relapsed within 12 months after allogeneic hematopoietic stem cell transplantation (allo-HSCT) were included. Step-wise dose ramp-up of blinatumomab was used to minimize initial CRS and neurotoxicities. Blinatumomab was infused at 9 µg/day for the first week, followed by 28 µg/day for the remaining 3 weeks. Treatment was cycled every 6 weeks. The results showed that CR/CRh was achieved in 43% of the patients (33% CR + 10% CRh) after the first two cycles. The median RFS was 5.9 m (median follow-up of 8.9 m), and the median OS was 6.1 m (median follow-up of 9.8 m) [38]. Among the 81 patients who achieved CR/CRh, 40% proceeded to allo-HSCT. Febrile neutropenia and anemia were common adverse events (AE). Severe CRS events including hypoxia, high fever and hypotension were reported in 3 patients. Tremors, seizure and mental status changes were reported as common neurotoxicity. It was noticed that 20% of the patients who received blinatumomab in this study were still alive after 2 years. Therefore, the data from this study were compared with historical data with long-term outcome from 1139 Ph negative B-ALL patients [47]. The long-term survival was estimated [47]. The estimated long-term (60 m) OS rate (12.4% vs 5.4%) and median OS (76.1 vs 38.6 m) of this clinical trial were significantly better than those from historical group before blinatumomab era. Even though these were not the results from a randomized study, these findings implied that blinatumomab has the potential to be better than salvage chemotherapy.

To confirm the efficacy of blinatumomab for R/R ALL, a phase III randomized trial (the TOWER trial, NCT 02013167) was done to compare blinatumomab versus salvage chemotherapy. This study enrolled 405 patients. The patients were randomized in a 2:1 ratio. 271 patients received blinatumomab, 124 patients received salvage chemotherapy. Compared with salvage chemotherapy, blinatumomab monotherapy had better OS (7.7 m vs 4.0 m, P = 0.01), CR rate (34% vs 16% in 12 weeks, P < 0.001) and EFS rate (31% vs 12% at 6 m, P < 0.001) in r/r B-ALL patients [48].

### Blinatumomab for MRD+ ALL

MRD remains measurable in 30 to 50% of adult ALL patients in hematologic CR after chemotherapy. It has been well established that positive MRD in ALL is associated with higher relapse rate and poor OS [17]. Allo-HSCT was reported to increase the 5-year RFS of MRD+ ALL from 11 to 44% [12].

An analysis of 20 evaluable ALL patients treated with blinatumomab revealed negative MRD in 16 of them, suggesting deep response in 80% patients [49]. The final analysis of this study reported a median follow-up of 50.8 months. Half of the 20 patients (50%) remained in CR 5 years after the initial treatment [50].

Blinatumomab was examined in MRD+ B-ALL patients in a multicenter open-label single-arm phase 2 study in patients with MRD+ (≥ 10^−3^) B-ALL who were in CR1 or CR 2/3. Among the 116 patients enrolled, 113 patients who received blinatumomab were evaluable. Among these, 78% were found to have negative MRD (MRD responders) after 1 cycle of blinatumomab. Compared with MRD non-responders, MRD responders had longer RFS (23.6 vs 5.7 months; P = 0.002) and OS (38.9 vs 12.5 months; P = 0.002). This study confirmed that blinatumomab is effective in eliminating MRD [51]. After a minimum follow-up of 3 years (median 53.1 m), OS has reached a plateau and the median survival has not been reached among the patients with a complete MRD response [52]. Blinatumomab became the first FDA-approved treatment for MRD + B-ALL in 2018 [53].

### Blinatumomab for Ph+ ALL

Clinical trials have been initiated to characterize the activity of blinatumomab in Ph+ R/R ALL patients. In a preliminary report, 45 patients were enrolled [54]. 36% (95% CI, 22% to 51%) CR/CRh were achieved. MRD negativity was 88% among the patients who achieved CR/CRh. 44% of the CR/CRh patients went on to receive allo-HSCT. This rate is consistent with the results from previous studies. Treatment emergent AEs (TEAE) were similar to those reported in other studies.

## Blinatumomab in combination therapy for ALL

### Blinatumomab + tyrosine kinase inhibitors

Tyrosine kinase inhibitors (TKI) are playing a major role in the therapy for Ph+ ALL [24, 26, 55, 56]. TKIs in combination with blinatumomab are being evaluated for Ph+ ALL [57]. Blinatumomab + ponatinib was used in 15 patients with relapsed Ph+ ALL [58]. In this retrospective analysis, ponatinib was given daily whereas blinatumomab was given a median of 3 cycles. 14 of the 15 patients achieved cytogenetic remission, with molecular CR in 12 patients. Two patients had CNS relapse while in molecular remission. The follow-up was short (median 8.5 months). A prospective trial is underway to evaluate the chemotherapy-free regimen (NCT03263572).

A group from the Memorial Sloan-Kettering Cancer Center reported a retrospective analysis of 11 patients who received blinatumomab plus one of the TKIs (ponatinib, dasatinib or nilotinib) [59]. Seven of the 11 MRD+ patients became MRD negative. The median follow-up was 7.7 months (range 3.2–16.0 months). Grade 1 CRS was seen in three patients and no patient had neurotoxicity. The blinatumomab and ponatinib combination was reported to have higher risk of liver enzyme abnormality.

Overall, the combination of blinatumomab with TKI was well tolerated. The chemotherapy-fee regimen appears to be promising to serve as a bridge therapy prior to allo-HSCT.

### Blintumomab + immune checkpoint inhibitors

Recently, a multi-center phase I dose-escalation study was initiated to evaluate the combination of blinatumomab with nivolumab and ipilimumab for R/R CD19+ ALL patients [60]. The patients were scheduled to receive up to 5 cycles of blinatumomab and 1 year of nivolumab/ipilimumab. The first part of the study was to evaluate the safety of combining blinatumomab with nivolumab. Once the dose is established, ipilimumab dose escalation will be added. A preliminary report enrolled 8 adults at dose level I. Five patients were evaluable. Common AEs included liver enzyme elevation and chemical pancreatitis. DLT was reported to be Infusion-related reactions to nivolumab. Four out of five evaluable patients achieved CR with -MRD. It appears that the blinatumomab/nivolumab combination in R/R ALL is well tolerated. The next phase of the study to add ipilimumab is ongoing.

### Blinatumomab + chemotherapy

Hyper-CVAD is a commonly used regimen for ALL therapy [61–66]. In an attempt to improve response rate and quality, blinatumomab was added to hyper-CVAD regimen in a phase 2 study [67]. After induction therapy with 4 cycles of Hyper-CVAD, blinatumomab was given as consolidation therapy for a total of 4 cycles. Monoclonal antibodies against CD20 were added for those patients with CD20+ ALL. In this study, blinatumomab was also added to the maintenance phase on cycles 4, 8 and 12 [57]. POMP (6-mercaptopurine, vincristine, methotrexate, prednisone) regimen was used for maintenance on cycles 1–3, 5–7, 9–11 and 13–15. A preliminary report of 17 patients revealed an ORR of 100%. MRD negativity was 93%. Among 14 evaluable patients, 9 patients have completed hyper-CVAD plus blinatumomab sequential therapy and entered maintenance phase. With a median follow-up of 14 months (range, 3–20 months) at the time of the report, the rates of OS and CR were 94% and 93%, respectively. A transient grade 3 CRS event was reported in one patient, and one had grade 3 neurotoxicity. These two patients both recovered after interruption of blinatumomab and prompt steroid therapy. Therefore, the sequential combination of Hyper-CVAD and blinatumomab was well tolerated as frontline regimen in B-ALL. This study attempts to reduce Hyper-CVAD chemotherapy to 4 cycles from conventional 8 cycles by adding 4 cycles of blinatumomab. This study also added blinatumomab in the maintenance phase so that the POMP maintenance time is reduced from usually 3 years to 12 months. In conclusion, this study may lead to reduction of chemotherapy toxicity and duration of maintenance therapy.

### miniHCVD + inotuzumab + blinatumomab

Inotuzumab ozogamicin (INO) is CD22 antibody–drug conjugate which has been approved for R/R ALL [61, 68–77]. Both INO and blinatumomab (blina) were superior as single agent to salvage chemotherapies in R/R ALL [37, 74]. Blinatumomab is being studied in combination with the miniHCVD-INO regimen for newly diagnosed ALL patients [71, 78–80]. Rituximab was added in patients with CD20 expression ≥20%. There were three phases in the therapy regimen: intensification, consolidation and maintenance. In the intensification phase, 4 cycles of miniHCVD was followed by 4 cycles of INO. Two lower doses of INO was given in each cycle. 4 cycles of blinatumomab was given in the consolidation phase. In th maintenance phase, 4 more cycles of blinatumomab were given on month 4, 8, 12 and 16. The POMP regimen was given in the maintenance as described above for a total of 12 cycles. Addition of blinatumomab makes it possible to reduce POMP maintenance cycles from 3 years to 12 months.

In a recent report, 58 newly diagnosed elderly B-ALL patients were enrolled [79]. Fifty-four patients were evaluable for morphological responses and 57 patients were evaluable for MRD status. Both ORR and MRD negativity were 95%. Sinus occlusive syndrome (SOS) was known to be associated with INO. SOS was seen in 8–11% of the patients. With a median follow-up of 28 months, the 3-year OS rate was estimated to be 54%. Therefore, it appears that adding blinatumomab to the miniHCVD + INO regimen was safe and effective in elderly patients with newly diagnosed ALL.

The miniHCVD + INO +/− blina regimen is also being studied in R/R ALL [78, 80]. In a recent report, 17 out of 84 patients received miniHCVD + INO + blina [80]. The SOS rate was markedly reduced to 0% from 15% after INO was split to two lower doses each cycle. Although the schedules are complicated, this low-intensity miniHCVD + INO + blina regimen appears to be well tolerated and effective in R/R ALL patients. The long interval between INO and allo-HSCT as well as split-dose INO has markedly reduced the SOS risk.

## Conclusion and future perspectives

Blinatumomab has been approved for patients with R/R B-ALL and MRD + B-ALL. Blinatumomab is being studied for use in frontline therapy of newly diagnosed B-ALL. Adding blinatumomab to the low intensity miniHCVD + INO regimen in the consolidation and maintenance phases appears to be promising. The mechanisms of blinatumomab resistance and predictive biomarkers for response remain uncertain [81]. Blinatumomab in maintenance therapy appears to be promising to minimize chemotherapy and reduce therapy duration. More BiTE antibodies are coming to clinical applications [82, 83]. New regimens incorporating blinatumomab may lead to new therapy modalities for ALL. Combination of blinatumomab with TKIs or with immune checkpoint inhibitors are ongoing and may result in chemotherapy-free regimens for ALL (Table 1).

**Table 1:** Ongoing clinical trials of combined treatment with blinatumomab for B-ALL

NCT Number	Patients	Treatment	Phase
NCT02877303	Newly diagnosed B-ALL	Blinatumomab + hyper-CVAD	Phase 2
NCT03367299	Untreated Ph− CD19+ B-ALL	Blinatumomab + chemotherapy	Phase 2
NCT03480438	Older newly diagnosed Ph/BCR-ABL- CD19+ B-ALL	Blinatumomab + chemotherapy	Phase 2
NCT03518112	Ph−R/R B-ALL	Blinatumomab + chemotherapy	Phase 2
NCT03914625	Newly diagnosed standard risk or down syndrome B-ALL and localized B-Lly	Blinatumomab + chemotherapy	Phase 3
NCT02143414	Older newly diagnosed Ph−B-ALL	Blinatumomab + chemotherapy	Phase 2
	Older newly diagnosed or R/R Ph+ (Ph-like) B-ALL	Blinatumomab + dasatinib + prednisone	
NCT03263572	Ph/BCR-ABL+ B-ALL	Blinatumomab + ponatinib + cytarabine + Methotrexate	Phase 2
NCT03147612	R/R Ph+/BCR-ABL + B-ALL	Blinatumomab + ponatinib + chemotherapy	Phase 2
NCT02744768	Newly diagnosed adult Ph+ B-ALL	Blinatumomab + dasatinib	Phase 2
NCT03605589	R/R B-ALL	Blinatumomab + pembrolizumab	Phase 1
NCT03160079	R/R B-ALL	Blinatumomab + pembrolizumab	Phase 1/2
NCT03512405	R/R B-ALL	Blinatumomab + pembrolizumab	Phase 1/2
NCT02879695	R/R B-ALL	Blinatumomab + nivolumab with or without ipilimumab	Phase 1
NCT02997761	R/R B-ALL	Blinatumomab + ibrutinib	Phase 2
NCT03739814	Newly diagnosed or R/R CD22+ B-ALL	Blinatumomab + inotuzumab ozogamicin	Phase 2
NCT03751709	R/R CD19+ B-ALL	Blinatumomab + HMCT	Phase 1
NCT03849651	Hematologic malignancies	Blinatumomab + DLI	Phase 2
NCT03982992	B-ALL with MC or MRD-positive after allo-HSCT	Blinatumomab + DLI	Phase 2
NCT02790515	R/R hematologic malignancies	Blinatumomab + haploidentical HCT	Phase 2

*R/R* relapsed/refractory; *B-ALL* B-cell acute lymphoblastic leukemia; *HMCT* HLA-mismatched cellular therapy; *BM* bone marrow; *B-Lly* B-Lymphoblastic lymphoma; *HCT* hematopoietic cell transplant; *DLI* donor lymphocyte infusion; *MC* mixed chimerism; *MRD* minimal residual disease; *allo-HSCT* allogeneic hematopoietic stem cell transplantation

## Data Availability

The material supporting the conclusion of this review has been included within the article.

## Abbreviations

CR: complete remission
B-ALL: B cell acute lymphoblastic leukemia
r/r: relapsed and refractory
SOC: standard of care
OS: overall survival
m: months
MRD: minimal residual disease
FDA: Food and Drug Administration
AEs: adverse effects
CRh: CR with partial hematologic recovery
allo-HSCT: allogeneic hematopoietic stem cell transplantation
CL: clearance
cIVi: continuous intravenous infusion
Css: steady-state concentrations
NEs: neurologic events
CRS: cytokine release syndrome
TKIs: tyrosine kinase inhibitors
DLI: donor lymphocyte infusions
